# Primary Cilia-Mediated Mechanotransduction in Human Mesenchymal Stem Cells

**DOI:** 10.1002/stem.1235

**Published:** 2012-09-11

**Authors:** David A Hoey, Shane Tormey, Stacy Ramcharan, Fergal J O'Brien, Christopher R Jacobs

**Affiliations:** a Department of Biomedical Engineering, Columbia University in the City of New YorkNew York, New York, USA; b Department of Anatomy, Royal College of Surgeons in IrelandDublin, Ireland; 3 Department of Mechanical, Aeronautical and Biomedical Engineering, Centre for Applied Biomedical Engineering Research, Materials and Surface Science Institute, University of LimerickLimerick, Ireland; d Trinity Centre for Bioengineering, Trinity College DublinDublin, Ireland

**Keywords:** Human mesenchymal stem cell, Primary cilium, Mechanotransduction, Fluid flow, Osteogenic Differentiation, Proliferation

## Abstract

Physical loading is a potent stimulus required to maintain bone homeostasis, partly through the renewal and osteogenic differentiation of mesenchymal stem cells (MSCs). However, the mechanism by which MSCs sense a biophysical force and translate that into a biochemical bone forming response (mechanotransduction) remains poorly understood. The primary cilium is a single sensory cellular extension, which has recently been shown to demonstrate a role in cellular mechanotransduction and MSC lineage commitment. In this study, we present evidence that short periods of mechanical stimulation in the form of oscillatory fluid flow (OFF) is sufficient to enhance osteogenic gene expression and proliferation of human MSCs (hMSCs). Furthermore, we demonstrate that the cilium mediates fluid flow mechanotransduction in hMSCs by maintaining OFF-induced increases in osteogenic gene expression and, surprisingly, to limit OFF-induced increases in proliferation. These data therefore demonstrate a pro-osteogenic mechanosensory role for the primary cilium, establishing a novel mechanotransduction mechanism in hMSCs. Based on these findings, the application of OFF may be a beneficial component of bioreactor-based strategies to form bone-like tissues suitable for regenerative medicine and also highlights the cilium as a potential therapeutic target for efforts to mimic loading with the aim of preventing bone loss during diseases such as osteoporosis. Furthermore, this study demonstrates a role for the cilium in controlling mechanically mediated increases in the proliferation of hMSCs, which parallels proposed models of polycystic kidney disease. Unraveling the mechanisms leading to rapid proliferation of mechanically stimulated MSCs with defective cilia could provide significant insights regarding ciliopathies and cystic diseases. Stem Cells*2012;30:2561–2570*

## INTRODUCTION

The formation and regeneration of bone tissue requires the concerted effort of numerous signals that result in the aggregation, proliferation, and differentiation of mesenchymal stem cells (MSCs) into bone forming osteoblasts [1]. A key signal regulating bone formation is physical loading. Loading-induced bending of bone generates strain gradients in the medullary cavity resulting in the development of hydrostatic pressure and fluid flow-induced shear stress, which directly stimulates MSCs within the marrow [2]. Such loading of rat tibiae has demonstrated enhanced bone formation through the proliferation and recruitment of osteoprogenitors within the marrow cavity [3–5] while unloading has been associated with decreased osteoblastogenesis [6, 7]. Osteoporosis is a debilitating bone disease, which occurs in part when MSCs fail to produce sufficient numbers of osteoblasts to counteract bone resorption by osteoclasts. Therefore, in order to combat such debilitating diseases, a greater understanding of the role of physical loading in regulating human MSC (hMSC) behavior is required. This would greatly aid in the development of mechanotherapies to prevent bone loss [8] in addition to advancing bioreactor-based bone tissue engineering strategies [9].

Direct mechanical stimulation of MSCs in vitro has demonstrated a strong role for mechanics in regulating stem cell behavior [10–12]. Several studies have explored the effect of fluid flow-induced shear stress on the osteogenic differentiation of MSCs whereby the application of fluid flow enhances the formation and maturation of extracellular matrix through the regulation of osteogenic genes and protein expression [13–18]. Only a few studies have investigated this effect in hMSCs, the majority of which have used long-term flow exposure in three-dimensional tissue engineering scaffolds. Scaglione et al. [19], Bjerre et al. [20, 21], and Zhao et al. [22] subjected hMSCs to perfusion flow for 10, 20, and 21 days respectively. Such flow regimens enhanced the expression of ALP, BMP2, BSP, OPN, and COL1α1. However, Tjabringa et al. [23] demonstrated that as little as 1 hour of pulsatile fluid flow was sufficient to enhance cyclo-oxygenase-2 (COX2) gene expression in adipose-derived hMSCs. Fluid flow resulting from activities which load the skeleton are predicted to be dynamic and oscillatory in nature yet only a small number of studies have investigated the effect of oscillatory fluid flow (OFF) on hMSC behavior. Li et al. [24] subjected hMSCs to 2 hours of OFF and found increases in OPN and OCLN gene expression in addition to an increase in proliferation. Furthermore, a series of studies by Riddle et al. [25, 26] demonstrated an increase in proliferation of hMSCs following high magnitude, short-term OFF. Due to the enhanced proliferative and osteogenic effect of fluid flow on hMSCs, flow perfusion bioreactors are becoming more significant in bone tissue engineering approaches, yet the effect of physiologically relevant OFF remains poorly understood [9].

Despite the potent role of physical loading in bone metabolism and, more specifically, stem cell differentiation, the mechanisms of mechanotransduction (translation of a biophysical force into a biochemical response) remain to be determined. Understanding these mechanisms may lead to novel approaches to promote bone formation through therapeutic intervention, mimicking the effect of physical loading at a molecular level. The primary cilium is a singular, immotile microtubule-based cellular extension, which projects from the apical surface of nearly every cell in the human body [27], and studies have recently demonstrated a role for the cilium in cellular mechanotransduction [28–30]. Although originally identified over a century ago [31, 32], recent advancements in imaging technology and the discovery of a link between defective cilia and numerous human pathologies [33] has fuelled research revealing the cilium to be a complex signaling center regulating key pathways such as hedgehog (HH) and Wnt [34]. For example, upon HH ligand binding of the Patched1 (PTCH1) receptor, PTCH1 derepresses Smoothened which in turn translocates to the tip of the primary cilium activating the Gli transcription factors [34]. In addition, through the specific localization of receptors and mechanosensitive channels to the ciliary axoneme, the cilium has demonstrated both a chemosensory and mechanosensory role across numerous tissues such as the kidney epithelium [35, 36], endothelium [37], liver [38, 39], cartilage [40] and, more recently by our lab and others, in bone [41–46]. It has been demonstrated that the more differentiated cells of the osteogenic lineage such as the osteoblast and osteocyte require a primary cilium for osteogenic responses to OFF [41] and mice with a bone-specific primary cilia knockout have inhibited bone formation due to loading [47]. Moreover, hMSCs require a primary cilium for biochemically induced differentiation [44]. However, to date, a role for the primary cilium in hMSC mechanotransduction remains undetermined.

As hMSCs hold great promise as potential treatments for bone loss diseases, either through direct transplantation in vivo or via bone tissue engineering strategies in vitro [48], this study aimed to elucidate the effects of physiologically relevant mechanical stimulation on stem cell behavior and elucidate the mechanism by which stem cells sense and transduce a mechanical stimulus into a biochemical response. In this study, we demonstrate that short periods of mechanical stimulation are sufficient to significantly enhance osteogenic gene expression and the proliferation of hMSCs. Furthermore, the study shows that the stem cell primary cilium is required for this mechanically-mediated increase in osteogenic gene expression and, surprisingly, is required to control and limit increases in proliferation. Collectively, these data demonstrate the importance of mechanical stimulation in regulating stem cell behavior, introduces a novel stem cell mechanotransduction mechanism via the primary cilium, and highlights the importance of the cilium in regulating stem cell mechanoresponses, further highlighting the cilium as a potential therapeutic target to mimic the effect of physical loading.

## MATERIALS AND METHODS

### hMSC Culture and Osteogenic Differentiation

MSCs harvested from human bone marrow were obtained from both Lonza (Lonza, Walkersville, MD, USA, http://www.lonza.com) and STEM CELL Technologies (STEMCELL, Vancouver, Canada, http://www.stemcell.com). hMSCs were maintained in growth media consisting of Dulbecco's modified Eagle's medium (DMEM) low glucose (Invitrogen, Carlsbad, CA, http://www.invitrogen.com) supplemented with 10% fetal bovine serum (FBS) and 1% penicillin-streptomycin (P/S). For gene expression studies, hMSCs were seeded onto fibronectin-coated glass slides at 175,000 cells per slide and maintained in growth media supplemented with 25 μM ascorbic acid 2-phosphate, 5 nM dexamethasone, and 5 mM β-glycerophosphate for 48 hours prior to the application of flow. These concentrations represent minimal levels for the support of osteogenic differentiation of hMSCs [49], thereby allowing greater scope to investigate the effect of a biophysical versus a biochemical stimulus on the differentiation of hMSCs. For biochemically induced osteogenic differentiation studies, cells were cultured in osteogenic induction media purchased from Invitrogen (Invitrogen). For proliferation rate studies, hMSCs were seeded onto fibronectin-coated glass slides at 100,000 cells per slide and were maintained in DMEM low glucose supplemented with 0.5% FBS and 1% P/S 24 hours prior to the application of a mechanical stimulus. All experiments were performed with passage four cells or less.

### Immunoctyochemistry

hMSCs were seeded onto fibronectin-coated glass slides at 100,000 cells per slide and maintained in DMEM low glucose containing 0.5% FBS (to arrest cell mitosis and allow maximal ciliogenesis) and 1% P/S for a minimum of 48 hours before processing for immunofluorescence. Cells were fixed in 10% neutral buffer formalin for 10 minutes. Fixed cells were rinsed three times with phosphate buffered saline (PBS), permeabilized with 0.1% Triton X-100 for 10 minutes, followed by a blocking wash consisting of 1% bovine serum albumin (BSA) for 2 hours. Cells were then incubated in monoclonal mouse anti-acetylated α tubulin (Abcam, Cambridge, U.K., http://www.abcam.com) diluted 1:1,500 in PBS containing 1% BSA for 24 hours at 4°C, followed by AlexaFluor (Invitrogen) 546 anti-mouse IgG diluted 1:200 in PBS containing 1% BSA for 1 hour at room temperature. Nuclei and the actin cytoskeleton were counterstained with 4′,6-diamidino-2-phenylindole (DAPI) (1 μg/ml) and Phalloidin (33 nM), respectively, both obtained from Invitrogen. To visualize the microtubule network within living hMSCs, 250 nM oregon green conjugated-taxol (Tubulin Tracker, Invitrogen) was added to cells for 30 minutes. Cells were then washed three times with PBS and imaged immediately. Oregon green conjugated-taxol does not alter cell or primary cilium morphology.

Images were captured using a Leica TCS SP5 laser scanning confocal microscope equipped with a ×100, 1.46 NA oil immersion objective. Fifteen representative images were captured per slide, and the total number of primary cilia was counted and compared to total number of nuclei. Three-dimensional reconstructions of z-stacks were generated to determine ciliary length and orientation.

### Application of OFF

OFF was applied to cells using a previously described parallel plate flow chamber [50]. In brief, OFF was driven by Hamilton glass syringes in series with rigid walled tubing and a parallel plate flow chamber. The syringe was mounted in and driven by a previously described custom-built mechanical loading device [50]. For gene expression studies, the flow rate was chosen to yield a peak shear stress of 1.0 Pa (28 ml/min). For proliferation rate studies, the flow rate was adjusted accordingly to yield a peak shear stress of both 1.0 Pa (28 ml/min) and 2.0 Pa (56 ml/min). For all experiments, cells were exposed to 2 hours of OFF.

### Small-Interfering RNA Transfection

The formation of functional primary cilia was inhibited by small-interfering RNA (siRNA)-mediated depletion of IFT88/Polaris. IFT88/Polaris is an intraflagellar transport protein (IFT) required for functional ciliogenesis [51]. hMSCs were transfected with 20 μM siRNA targeting IFT88/Polaris (sequence 5′-AAUAGCAUCUGAAUACUGACCAGCC-3′-) or with a scrambled siRNA for 8 hours using Lipofectamine 2000 (Invitrogen). HMSCs were maintained in growth media for a further 72 hours before the application of a mechanical stimulus. Neither siRNA targeting Polaris nor scrambled siRNA had any effect of cellular morphology.

### Proliferation

5-Ethynyl-2′-deoxyuridine (EdU) incorporation (Click-iT EdU proliferation kit, Invitrogen) was used to determine the proliferation rate of hMSCs. EdU is a nucleoside analog of thymidine and is incorporated into DNA during active DNA synthesis. Upon cessation of flow, cells were incubated in DMEM low glucose containing 0.5% FBS and 1% P/S for 20 hours followed by 1-hour incubation in the same media supplemented with a 10 μM EdU solution. Cells were then processed for immunofluorescence as above. EdU incorporation was detected using an Alexa Flour 488 azide, and cell number was determined using DAPI as above. Fifteen representative images were captured per slide using an Olympus CKX41 inverted microscope fitted with a ×10 objective (40 cells per image), and the total number of EdU positive cells was compared to total cell number.

### Real-Time Quantitative Polymerase Chain Reaction

Total RNA was isolated using Tri-Reagent (Invitrogen) according to the manufacturer's instructions. The 260/280 absorbance ratio was measured for verification of the purity and concentration of RNA. Two to three microgram of RNA was used for reverse transcription using the high capacity cDNA reverse transcription kit (Applied Biosystems, Foster City, CA, http://www.appliedbiosystems.com). Primers and probes for COX2, BMP2, RUNX2, OSTERIX, OPN, OCLN, ALKLP, COL1α1, BSP, POLARIS, PTCH1, GLI1, and GAPDH were obtained from Applied Biosystems. cDNA samples were then amplified by real-time quantitative polymerase chain reaction (qRT-PCR). Amplification curves for all genes were recorded, and the relative levels between samples were quantified using the relative standard curve method (ABI Prism 7700, Applied Biosystems). All samples were normalized to endogenous control GAPDH mRNA. All samples and standards were run in triplicate.

### Statistical Analysis

Statistical analyses were performed using GraphPad Prism (Version 5.0) software. All data are expressed as means ± SEM. Two-way ANOVA was used for analysis of variance with Bonferroni post-tests to compare between groups. For two sample comparisons, a Student's *t* test was used. Significance is indicated on figures as follows: *, *p* ≤ .05; **, *p* ≤ .01; ***, *p* ≤ .001.

## RESULTS

### Short Periods of Mechanical Stimulation Significantly Upregulate Osteogenic Gene Expression in hMSCs

**Figure 1:**
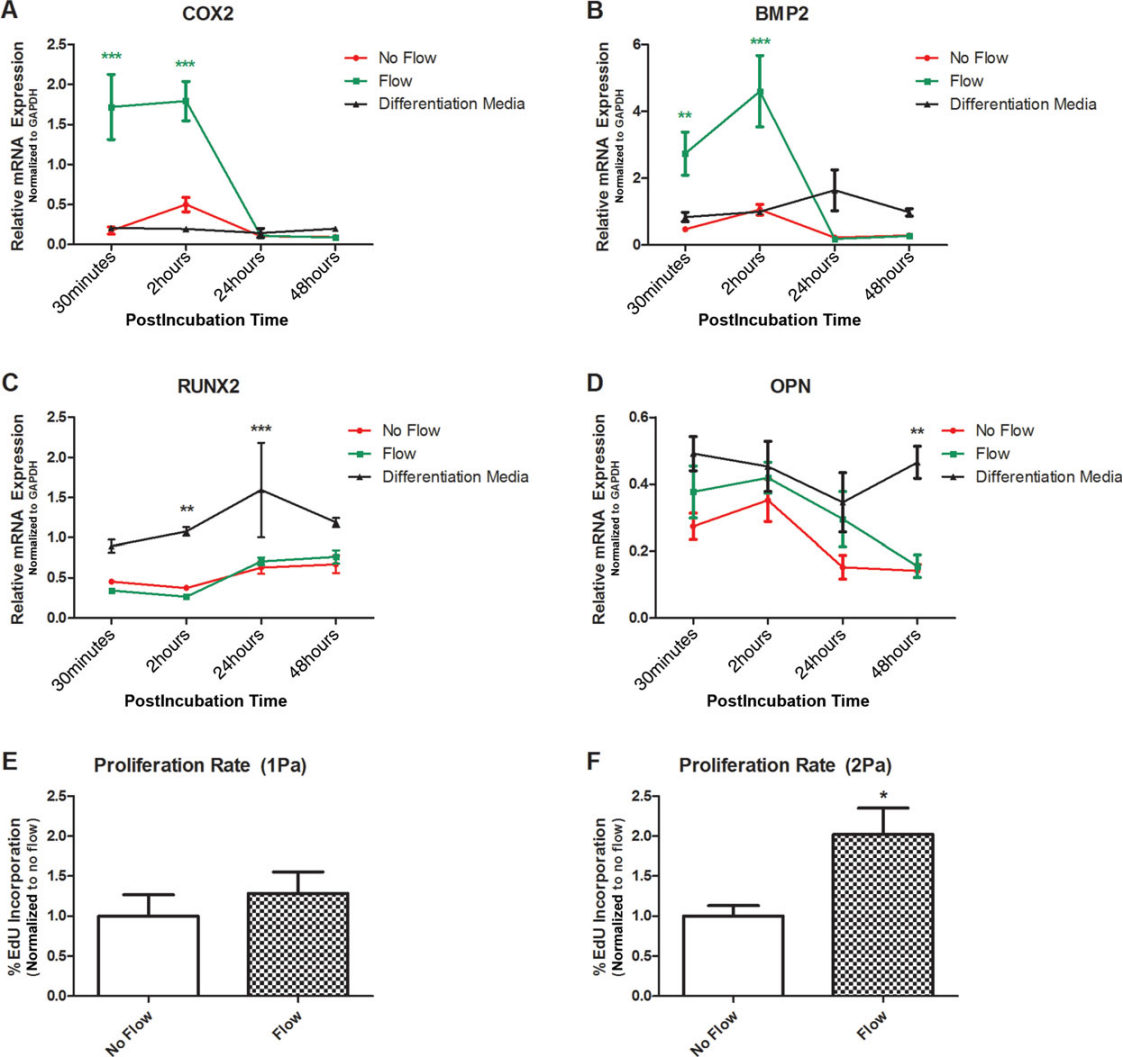
Effect of 2 hours of oscillatory fluid flow-induced 1 Pa of shear stress on the mRNA expression of **(A)** COX2, **(B)** BMP2, **(C)** RUNX2, and **(D)** OPN at 30 minutes, 2 hours, 24 hours, and 48 hours post-cessation of flow. Cells were cultured statically and in osteogenic differentiation media as controls. Furthermore, the effect of 2 hours of oscillatory flow-induced **(E)** 1 Pa and **(F)** 2 Pa of shear stress on the proliferation rate of human mesenchymal stem cells as assayed by 1 hour of EdU incorporation 20 hours post-cessation of flow. Mean ± SEM, * indicates significantly different than controls (*, *p* < .05; **, *p* < .01; ***, *p* < .001). Abbreviations: COX2, cyclo-oxygenase-2; EdU, 5-ethynyl-2′-deoxyuridine.

To investigate the effect of mechanical stimulation on the osteogenic gene expression of hMSCs, cells were subjected to 2 hours of OFF, and the mRNA expression of nine bone-related markers were assayed at 30 minutes, 2 hours, 24 hours, and 48 hours post-cessation of flow. The relative mRNA expression was compared to no-flow controls and cells cultured in osteogenic differentiation media. This short period of mechanical stimulation resulted in a significant, transient increase in COX2 and BMP2 mRNA expression over both static controls and osteogenic differentiation media controls within 30 minutes post-flow, which remained elevated until 2 hours post-flow before returning to basal levels thereafter (Fig. 1A, 1B). Surprisingly, mechanical stimulation did not significantly affect the mRNA expression of RUNX2, OPN, OCLN, ALKLP, COL1α1, or BSP at any time point. OSTERIX could not be reliably detected using qRT-PCR (Fig. 1C, 1D; Supporting Information Fig. S1A–S1D). The treatment of hMSCs with osteogenic differentiation media resulted in a significant increase in RUNX2 and OPN mRNA levels (Fig. 1C, 1D). Therefore, these data demonstrate that the application of short periods of OFF is sufficient to significantly enhance osteogenic gene expression in hMSCs.

### Short Periods of High Magnitude Mechanical Stimulation Enhances the Proliferation Rate of hMSCs

Mechanically-induced changes in hMSC proliferation was investigated by subjecting cells to two different magnitudes of OFF for 2 hours followed by EdU incorporation for 1 hour at 20 hours post-cessation of flow. Subjecting hMSCs to the same magnitude of mechanical stimulation used in the gene expression analysis (1 Pa; 28 ml/min) did not significantly alter the proliferation rate. However, increasing the magnitude of flow by a factor of two significantly increased the proliferation rate twofold (*p* < .05) over no-flow controls (Fig. 1E, 1F) demonstrating that higher magnitudes of flow are required to elicit a proliferative response in hMSCs.

### Primary Cilia Project Outward from the Surface of hMSCs at High Incidence and Cilium Formation Is Inhibited Following siRNA Transfection Targeting POLARIS

**Figure 2:**
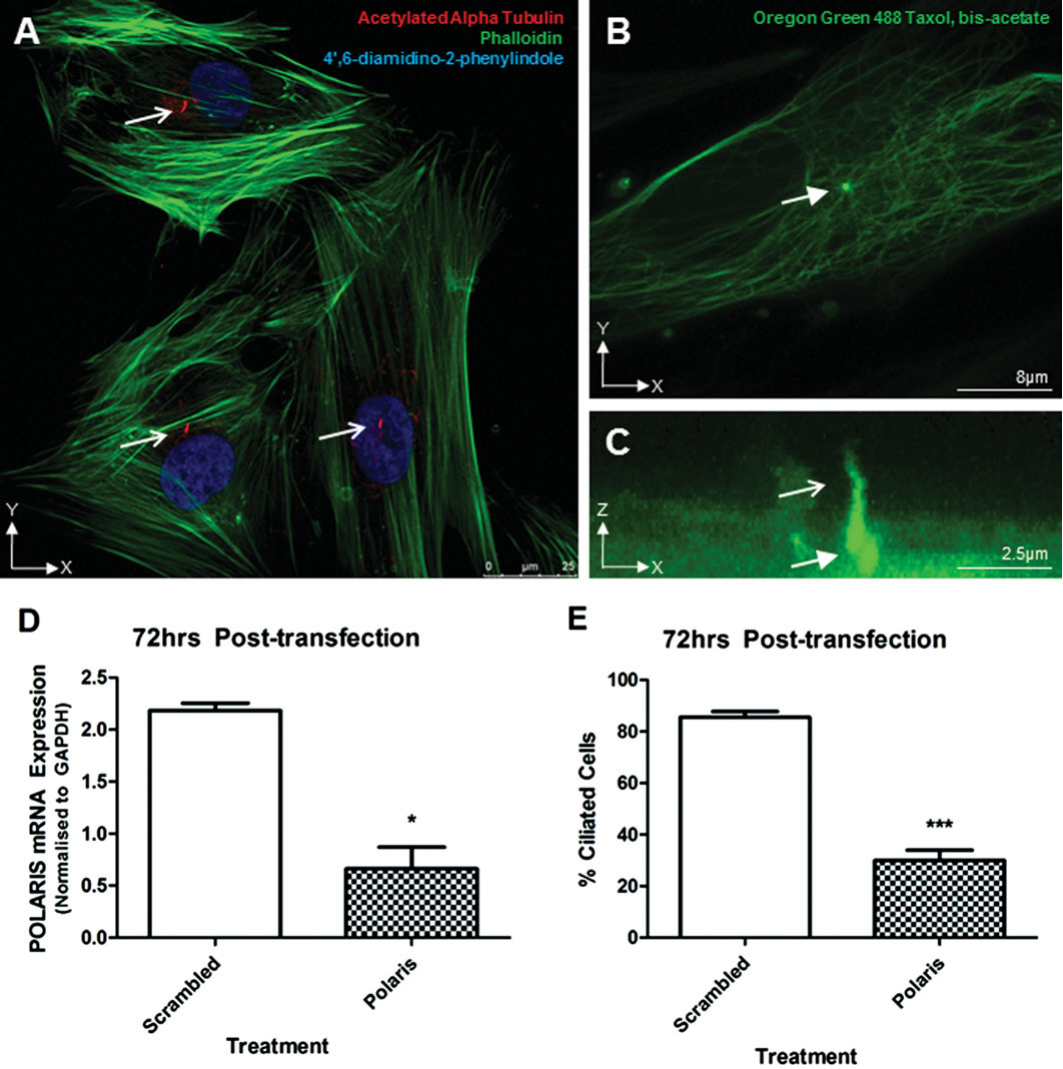
Confirmation of the existence of primary cilia on human mesenchymal stem cells (hMSCs). **(A):** Primary cilia stained with antiacetylated α-tubulin were present on 86% of hMSCs in the perinuclear region of the cell. **(B, C):** Live microtubule imaging demonstrated that hMSC primary cilia are intricately connected to the microtubule cytoskeleton and extend from the mother centriole 4–6 μm into the extracellular space. Open arrowhead: primary cilia; close arrowhead: centrioles. **(D):** Polaris mRNA expression of hMSCs and (C) number of ciliated cells 72 hours following transfection with scrambled small-interfering RNA (siRNA) and siRNA targeting Polaris. Mean ± SEM, * indicates significantly different than scrambled control (*, *p* < .05; ***, *p* < .001). Abbreviation: DAPI, 4′,6-diamidino-2-phenylindole.

The presence, orientation, and incidence of primary cilia on hMSCs were determined using both immunocytochemistry and live microtubule staining. Primary cilia were visualized as rod-like structures extending linearly from the perinuclear region of the cell on 86% ± 6% of hMSCs imaged. Live microtubule imaging revealed the cilium to be tightly connected with the microtubule cytoskeleton, extending 4–6 μm in length from the mother centriole out into the extracellular space (Fig. 2; Supporting Information Fig. S2).

**Figure 3:**
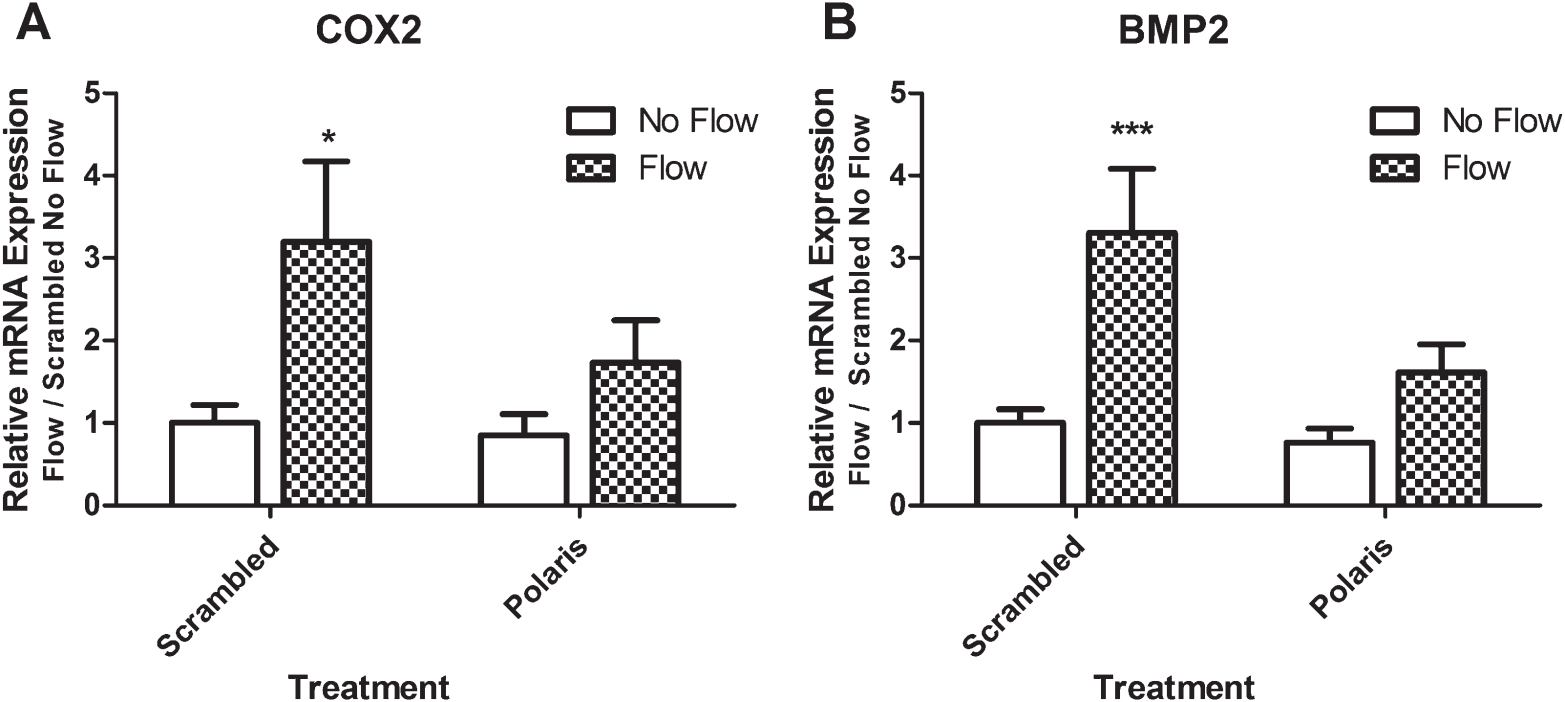
Effect of 2 hours of oscillatory fluid flow-induced 1 Pa of shear stress on the mRNA expression of **(A)** COX2 and **(B)** BMP2 2 hours post-cessation of flow in human mesenchymal stem cells transfected with scrambled small-interfering RNA (siRNA) and siRNA targeting POLARIS. Mean ± SEM, * indicates significantly different than no flow control (*, *p* < .05; ***, *p* < .001). Abbreviation: COX2, cyclo-oxygenase-2.

To prevent ciliogenesis, hMSCs were transfected with siRNA targeting POLARIS. 72 hours following transfection, mRNA levels of POLARIS were significantly reduced by 70% (*p* < .05). This reduction in mRNA levels has previously been shown by our lab to result in a corresponding decrease in protein levels [44]. To confirm the inhibition of ciliogenesis, primary cilium incidence was quantified using immunofluorescence 72 hours following transfection. The inhibition of POLARIS message resulted in a significant 65% reduction (*p* < .001) in the number of hMSCs which possessed a primary cilium (Fig. 2).

### Primary Cilia Are Required for the Upregulation of Osteogenic Genes in Response to Mechanical Stimulation in hMSCs

To investigate the role of the primary cilium in mechanically mediated changes in osteogenic gene expression, cells were subjected to 2 hours of 1 Pa of OFF, and COX2 and BMP2 mRNA expression were assayed 2 hours post-flow based on the findings from Figure 1. In cells treated with scrambled siRNA, exposure to flow 72 hours following siRNA transfection resulted in a significant 3.2-fold (*p* < .05) increase in COX2 and 3.3-fold (*p* < .001) increase in BMP2 mRNA expression over static controls. This increase correlates with previous findings (Fig. 1) demonstrating that the siRNA transfection alone did not influence the response to mechanical stimulation. However, hMSCs treated with siRNA targeting POLARIS followed by mechanical stimulation did not result in a significant increase in either COX2 or BMP2 mRNA expression (Fig. 3). Furthermore, static controls in both scrambled and POLARIS siRNA groups were not significantly different indicating that the presence of a primary cilium affects the cells ability to sense a mechanical stimulus and does not directly affect basal osteogenic gene expression at the time points investigated in this study.

### The Application of Mechanical Stimulation Inhibits HH Signaling in hMSCs and This Response Is not Dependent on the Primary Cilium

**Figure 4:**
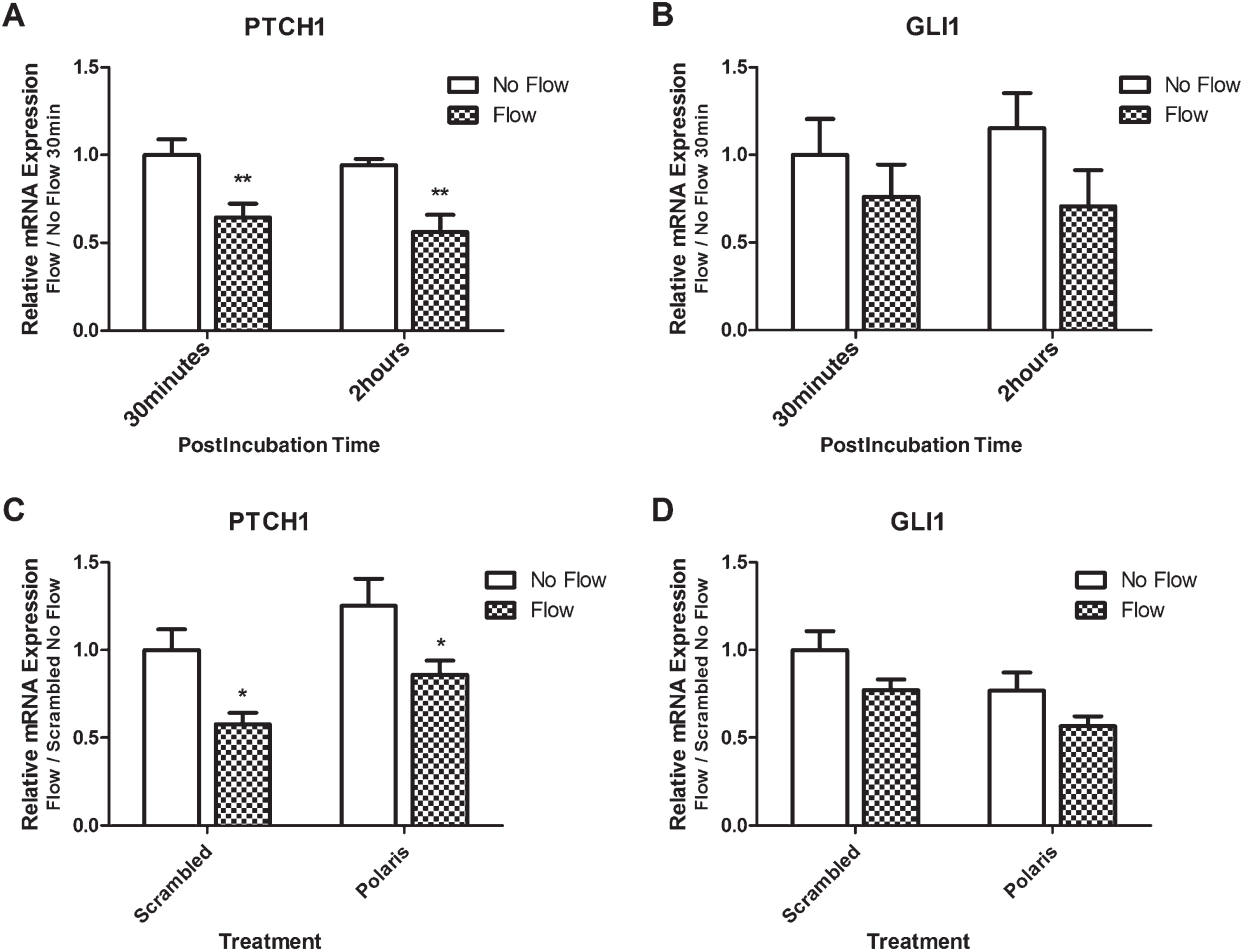
Effect of oscillatory fluid flow-induced 1 Pa of shear stress on the mRNA expression of **(A)** PTCH1 and **(B)** GLI1 30 minutes and 2 hours post-cessation of flow. Effect of oscillatory fluid flow-induced 1 Pa of shear stress on the mRNA expression of **(C)** PTCH1 and **(D)** GLI1 2 hours post-flow in human mesenchymal stem cells transfected with scrambled small-interfering RNA (siRNA) and siRNA targeting POLARIS. Mean ± SEM, * indicates significantly different than no flow control (*, *p* < .05; **, *p* < .01). Abbreviation: PTCH1, Patched1.

As the HH signaling pathway has been shown to be involved in the osteogenic differentiation of MSCs and is regulated by the primary cilium, we investigated the activity of this pathway in response to mechanical stimulation. The expression of the PTCH1 gene was significantly downregulated in response to 2 hours of OFF at both 30 minutes (*p* < .01) and 2 hours (*p* < .01) post-flow (Fig. 4A, 4B). A similar trend was witnessed in GLI1 expression, although the decrease was not significant. This decrease in HH signaling activity inversely parallels with the increase in COX2 and BMP2 gene expression witnessed at the same time points post-flow.

**Figure 5:**
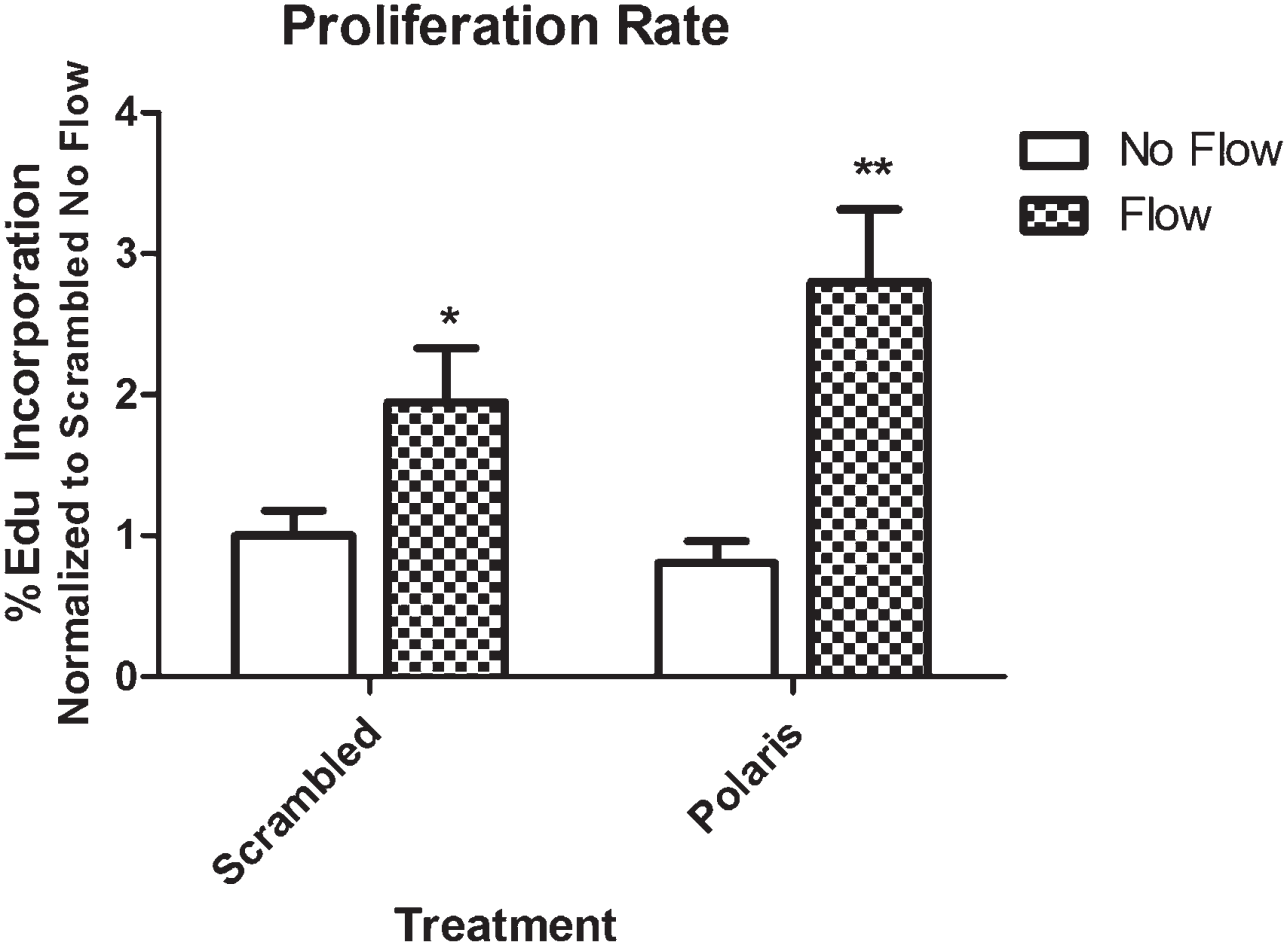
Effect of 2 hours of oscillatory fluid flow-induced 2 Pa of shear stress on the proliferation rate of human mesenchymal stem cells transfected with scrambled small-interfering RNA (siRNA) and siRNA targeting POLARIS. Mean ± SEM, * indicates significantly different than no flow control (*, *p* < .05; **, *p* < .01). Abbreviation: EdU, 5-ethynyl-2′-deoxyuridine.

To investigate whether the primary cilium was required for this mechanically mediated change in HH signaling, the expression of PTCH1 and GLI1 was assayed following 2 hours of OFF in hMSCs following transfection with scrambled siRNA and siRNA targeting Polaris. HMSCs which do not possess a primary cilium still displayed an inhibition of HH signaling (*p* < .05) following mechanical stimulation indicating that the cilium is not required for this response. In addition, as osteogenic gene expression is inhibited following removal of the cilium, it indicates that the HH signaling pathway is not directly involved in the inhibited osteogenic response to flow following cilia removal.

### HMSCs Which do not Possess a Primary Cilium Are More Responsive to the Proliferative Stimulus of High Magnitude OFF

To determine the role of the primary cilium in mechanically mediated changes in proliferation, hMSCs were subjected to 2 hours of OFF (2 Pa; 56 ml/min) following treatment with scrambled and siRNA targeting POLARIS, and EdU incorporation was assayed 20 hours post-flow. HMSCs transfected with scrambled siRNA demonstrated a similar 1.9-fold increase (*p* < .05) in the proliferation rate demonstrating again that the transfection process did not affect cell behavior. However, in cells treated with siRNA targeting Polaris, exposure to flow resulted in an even greater 2.8-fold (*p* < .01) increase in the proliferation rate of hMSCs (Fig. 5(. There was no significant difference in static proliferation rates between the scrambled and POLARIS targeting siRNA groups.

## DISCUSSION

The significance of the physical environment in regulating stem cell behavior has only recently come to be appreciated. This is particularly evident in bone, where physical loading is a key regulator of bone adaptation through the activation of osteoprogenitors during development [52], in adult tissue in vivo [3] and regenerative medicine strategies in vitro [10]. In order to fully capitalize on the positive effect of physical loading, a greater understanding of how this signal is transduced into an osteogenic response is required. In this study, we demonstrate that physical loading in the form of OFF-induced shear stress is a pro-osteogenic stimulus for hMSCs. In addition, we demonstrate for the first time that osteoprogenitors use the primary cilium in the mechanotransduction of fluid flow by mediating loading-induced changes in osteogenic gene expression and proliferation. Our findings therefore highlight OFF as a potential component of bioreactor-based strategies to form bone-like tissues suitable for regenerative medicine and furthermore highlight the primary cilium as a potential therapeutic target for efforts to mimic loading in vivo with the aim of preventing bone-loss during diseases such as osteoporosis.

Short periods of mechanical stimulation significantly enhanced osteogenic gene expression and the proliferation rate of hMSCs. Although the vast majority of studies to date have investigated the effect of long-term exposure of mechanical stimulation in MSCs [19–22], this study demonstrates that as little as 2 hours of OFF was sufficient to significantly upregulate COX2 and BMP2 mRNA over static and osteogenic differentiation media controls and remained upregulated for 2 hours following the cessation of flow. COX2 is an enzyme that catalyzes the synthesis of prostaglandin-E2 (PGE2). COX2, through the production of PGE2, mediates loading-induced bone formation and is critically involved in bone fracture repair in vivo [23, 53–55]. COX2 knockout mice display a marked reduction in osteoblastogenesis which correlates with significantly reduced levels of OSTERIX and RUNX2 gene expression, two pivotal early transcription factors in the osteogenic lineage. Interestingly, PGE2 and BMP2 treatment rescue this defect and enhance the expression of both transcription factors, indicating a role for BMP2 in osteoblastogenesis downstream of COX2 yet upstream of OSTERIX and RUNX2 [54]. Therefore, this study captured very early-stage markers of osteogenic differentiation. It must be noted that despite increases in osteogenic gene expression in response to flow, due to the transient nature of their expression, the duration and magnitude of flow used in this study may not be sufficient to fully drive the osteogenic lineage commitment of hMSC. Therefore, longer periods of mechanical stimulation and/or greater postincubation periods may be required to observe changes in the other markers assayed and verify osteogenic differentiation. In addition, only high magnitude mechanical stimulation generated a significant increase in the proliferation rate of hMSCs. This data is consistent with a series of studies by Riddle et al. where high flow rate OFF resulted in the release of ATP which acted in an autocrine/paracrine manner to enhance proliferation [25, 26]. ATP-induced proliferation activates phosphoinositide 3-kinase (PI3K)/Akt-, mTOR (mammalian target of rapamycin)/p70/S6K-, and ERK1/2-dependent signaling pathways in fibroblasts [56], all of which have been implicated in mechanically-mediated changes in hMSC and osteoblast proliferation [26, 57]. Therefore, numerous pathways may regulate mechanically-mediated changes in MSC proliferation via ATP-mediated purinergic signaling. As loading-induced bone formation is magnitude dependent [58], it is interesting to speculate that higher magnitude loading such as 2Pa shear represents a mechanosensing threshold whereby, in order to meet the bodies demands of greater bone formation, osteoprogenitor proliferation is initiated to provide sufficient numbers of bone forming cells. Although determining this is beyond the scope of this study, it is evident that OFF is a potent stimulus regulating hMSC behavior. In summary, short periods of OFF significantly enhanced osteogenic gene expression and proliferation of hMSCs and therefore supports the use of OFF as an effective component of bioreactor based tissue engineering strategies.

Primary cilia were shown to protrude 4-6 μm in length from the apical surface of hMSCs at high incidence. This high incidence is consistent with previous findings in hMSCs and human embryonic stem cells [44, 59, 60] and is considerably higher than what has been detected in more differentiated cells of the osteogenic lineage [41, 59] suggesting a greater role for the primary cilium in the progenitor cell. For example, during development the HH signaling pathway, which is regulated by the primary cilium, is known to play pivotal roles in the commitment of different lineages and it has been shown that stem cells which do not possess a primary cilium have a reduced capacity for neurogenesis [61] and cardiogenesis [62] due to impaired HH signaling. In addition to cilium incidence, primary cilia were shown to extend from the mother centriole, which is intimately connected with the cells cytoplasmic microtubule network, into the extracellular space. These observations suggest that stem cell primary cilia possess physical characteristics consistent with extracellular-sensing in more differentiated cells such as the osteoblast and osteocyte [41].

hMSCs which do not possess a primary cilium display an inhibited osteogenic response to mechanical stimulation demonstrating an important role for the cilium in regulating stem cell mechanotransduction. Flow-mediated increases in COX2 and BMP2 mRNA expression is lost in hMSCs with removal of the primary cilium yet the basal expression of these genes remained unaltered. This inhibition of osteogenic gene expression would not only suppress osteogenic differentiaiton in cells exposed to OFF but also through the potential loss of PGE2 (through COX2) and BMP2 secretion may directly affect the differentiation of adjacent cells throughout the stem cell niche. Therefore, these data point to a pro-osteogenic mechanosensory role for the primary cilium in hMSCs. Several mechanosensitive pathways regulated by the primary cilium have been shown to be important in the osteogenic differentiation of stem cells. Interestingly, a recent study demonstrated that activation of the HH signaling pathway is inversely correlated with osteogenic differentiation in hMSCs [59] despite a strong positive correlation in murine cells [27, 40, 63, 64]. In this study, the application of mechanical stimulation, which was sufficient to enhance osteogenic gene expression, resulted in an inhibition of HH signaling as measured by PTCH1 and GLI1 mRNA expression. These data therefore suggest that fluid flow counters HH suppression of osteogenic differentiation in hMSCs. Surprisingly, the flow-mediated inhibition of HH was not lost with removal of the primary cilium, indicating that HH signaling is not playing a direct role in our studies findings. Further work is required to fully elucidate the role of HH signaling in mechanically mediated osteogenic differentiation of hMSCs. Despite many cell types using the cilium in a mechanosensory role, it is becoming apparent that the underlying molecular mechanism differs depending on the cell type. For example, cilia-mediated mechanosensing in kidney epithelial cells is dependent upon intracellular calcium while in osteocytes cAMP is the second messenger molecule. It is distinctly possible that the molecular mechanism of cilia-based mechanosensing is preserved throughout the osteogenic lineage (MSC–osteoblast–osteocyte). Therefore, that would indicate AC6/cAMP signaling as the molecular mechanism [43]. In fact, the application of 30 minutes of OFF was sufficient to significantly enhance cAMP production in hMSCs (data not shown). Future work aims to delineate the exact molecular mechanosensing components involved in cilia-mediated hMSC mechanotransduction, the identification of which could allow direct pharmaceutical manipulation, resulting in therapeutic treatments for bone loss in diseases such as osteoporosis.

Unexpectedly, inhibiting primary cilia formation and function significantly enhanced flow-mediated hMSC proliferation, yet once again did not affect basal proliferation. Therefore, it seems that the cell uses the primary cilium to control mechanically mediated changes in proliferation. A similar phenomenon has been reported in epithelial cells of the kidney where defects in the primary cilium results in uncontrolled proliferation characterized by high mTOR activity, cyst formation, and ultimately in polycystic kidney disease (PKD). mTOR is a kinase belonging to the PI3K-related kinase family of proteins and has essential roles in protein translation, cell growth, and proliferation [65] and is activated in several types of tumors [66]. Boehlke et al. [67] recently demonstrated that bending of the primary cilium under flow results in the downregulation of mTOR activity. Shillingford et al. [68] showed that the ciliary protein, polycystin-1 (PC1), forms a complex with mTOR also inhibiting its activity and subsequently cell proliferation. This has led to speculation that defects in the primary cilium and/or PC1 leaves mTOR in an uncontrolled state, where it is susceptible to activation from other kinases such as ERK1/2 [69] which is known to be phosphorylated in hMSCs in response to OFF [26]. In effect, this would hypersensitize the cell to a pro-proliferative stimulus such as fluid flow. Our data supports this model indicating that this phenomenon is not tissue specific and therefore has far reaching significance as many ciliopathies such as PKD are characterized by uncontrolled proliferation and cyst formation.

Some limitations of this study should be mentioned. The primary cilium was required for OFF-mediated increases in early osteogenic gene expression, but the role of cilium in regulating OFF-mediated osteogenic lineage commitment at later time points was not verified. Although the data presented strongly indicate a role for the cilium in this response, further work is necessary to fully characterize the role of the cilium in mechanically-mediated stem cell differentiation. Inhibition of primary cilia formation was achieved by siRNA knockdown of Polaris. Although siRNA treatment significantly reduced primary cilium formation, 30% of hMSCs imaged post-transfection still possessed a primary cilium demonstrating that this technique is not completely effective in removing the primary cilium. However, a 65% reduction in cilium incidence was sufficient to significantly blunt hMSC responsiveness to fluid flow and therefore demonstrates this organelles role in hMSC mechanotransduction. Previous studies have used chloral hydrate treatment to remove cilia in parallel with siRNA. Chloral hydrate acts to disrupt the cilia/basal body connection but has also demonstrated nonspecific effects on cell behavior. Given that previous studies have not demonstrated different outcomes between the two methods and the potential nonspecific effects of a chemical treatment, chloral hydrate was not used in this study. Although this study demonstrates a role for the primary cilium in hMSC mechanotransduction, it is important to acknowledge that there are numerous other mechanosensitive organelles/molecules, which the stem cell could use for mechanotransduction. For example, adult bone cells which do not possess β1 integrin and/or focal adhesion kinase do not respond to fluid shear with an increase in osteogenic gene expression [70, 71]. Depending on the form, frequency or magnitude of stimulation the cell may use different mechanotransduction mechanisms, or, intriguingly, in some cases there may be dependent mechanisms. This may indeed be the case in tissues where β1 integrins have been shown to localize to the primary cilium, including chondrocytes [72] and kidney epithelial cells [73]. Although the investigation of this possible crosstalk is beyond the scope of this study, future work aims to explore other potential mechanisms of mechanotransduction in hMSCs and their potential involvement in cilia mediated mechanotransduction.

## CONCLUSION

This study presents evidence that the primary cilium plays a role in hMSC mechanotransduction, providing a novel mechanism by which a mechanical stimulus is translated into an osteogenic response in hMSCs. Based on these findings, the application of OFF may be a beneficial component of a bioreactor-based strategy to form bone-like tissues suitable for regenerative medicine and also highlights the primary cilium as a potential therapeutic target for efforts to mimic loading with the aim of preventing bone loss during diseases such as osteoporosis. Furthermore, this study demonstrates a role for the primary cilium in controlling mechanically mediated increases in the proliferation of hMSCs. Unraveling the mechanisms leading to rapid proliferation of MSCs with defective primary cilia could have far reaching implications for ciliopathies and the function of primary cilia in general.

## Supplementary material

Figure S1: Effect of 2hrs of oscillatory flow induced 1Pa of shear stress on the mRNA expression of (A) OCLN, (B) AKLP, (C) COL1α1, and (D) BSP at 30mins, 2hrs, 24hrs, and 48hrs post cessation of flow. Cells were cultured statically and in osteogenic differentiation media as controls. Mean ± SEM, * indicates significantly different than other groups (* P<0.05).

Figure S2: 3D rotational animation of a hMSC stained with anti-acetylated α-tubulin (primary cilium) and phalloidin (actin cytoskeleton). Red – primary cilium, green – actin cytoskeleton.

## References

[1] Corral DA, Amling M, Priemel M (1998). Dissociation between bone resorption and bone formation in osteopenic transgenic mice. Proc Natl Acad Sci USA.

[2] Gurkan UA, Akkus O (2008). The mechanical environment of bone marrow: A review. Ann Biomed Eng.

[3] Turner CH, Owan I, Alvey T (1998). Recruitment and proliferative responses of osteoblasts after mechanical loading in vivo determined using sustained-release bromodeoxyuridine. Bone.

[4] Rubin CT, Capilla E, Luu YK (2007). Adipogenesis is inhibited by brief, daily exposure to high-frequency, extremely low-magnitude mechanical signals. Proc Natl Acad Sci USA.

[5] David V, Martin A, Lafage-Proust MH (2007). Mechanical loading down-regulates peroxisome proliferator-activated receptor gamma in bone marrow stromal cells and favors osteoblastogenesis at the expense of adipogenesis. Endocrinology.

[6] Barou O, Palle S, Vico L (1998). Hindlimb unloading in rat decreases preosteoblast proliferation assessed in vivo with BrdU incorporation. Am J Physiol.

[7] Wronski TJ, Morey-Holton ER, Doty SB (1987). Histomorphometric analysis of rat skeleton following spaceflight. Am J Physiol.

[8] Khan KM, Scott A (2009). Mechanotherapy: How physical therapists' prescription of exercise promotes tissue repair. Br J Sports Med.

[9] McCoy RJ, O'Brien FJ (2010). Influence of shear stress in perfusion bioreactor cultures for the development of three-dimensional bone tissue constructs: A review. Tissue Eng Part B, Rev.

[10] Potier E, Noailly J, Ito K (2010). Directing bone marrow-derived stromal cell function with mechanics. J Biomech.

[11] Castillo AB, Jacobs CR (2010). Mesenchymal stem cell mechanobiology. Curr Osteoporosis Rep.

[12] Kelly DJ, Jacobs CR (2010). The role of mechanical signals in regulating chondrogenesis and osteogenesis of mesenchymal stem cells. Birth Defects Res C Embryo Today.

[13] Kreke MR, Huckle WR, Goldstein AS (2005). Fluid flow stimulates expression of osteopontin and bone sialoprotein by bone marrow stromal cells in a temporally dependent manner. Bone.

[14] Bancroft GN, Sikavitsas VI, van den DolderJ (2002). Fluid flow increases mineralized matrix deposition in 3D perfusion culture of marrow stromal osteoblasts in a dose-dependent manner. Proc Natl Acad Sci USA.

[15] McGarry JG, Klein-Nulend J, Prendergast PJ (2005). The effect of cytoskeletal disruption on pulsatile fluid flow-induced nitric oxide and prostaglandin E2 release in osteocytes and osteoblasts. Biochem Biophys Res Commun.

[16] Keogh MB, Partap S, Daly JS (2011). Three hours of perfusion culture prior to 28 days of static culture, enhances osteogenesis by human cells in a collagen gag scaffold. Biotechnol Bioeng.

[17] Knippenberg M, Helder MN, Doulabi BZ (2005). Adipose tissue-derived mesenchymal stem cells acquire bone cell-like responsiveness to fluid shear stress on osteogenic stimulation. Tissue engineering.

[18] Liu L, Yuan W, Wang J (2010). Mechanisms for osteogenic differentiation of human mesenchymal stem cells induced by fluid shear stress. Biomech Model Mechanobiol.

[19] Scaglione S, Wendt D, Miggino S (2008). Effects of fluid flow and calcium phosphate coating on human bone marrow stromal cells cultured in a defined 2D model system. J Biomed Mater Res. Part A.

[20] Bjerre L, Bunger C, Baatrup A (2011). Flow perfusion culture of human mesenchymal stem cells on coralline hydroxyapatite scaffolds with various pore sizes. J Biomed Mater Res. Part A.

[21] Bjerre L, Bunger CE, Kassem M (2008). Flow perfusion culture of human mesenchymal stem cells on silicate-substituted tricalcium phosphate scaffolds. Biomaterials.

[22] Zhao F, Chella R, Ma T (2007). Effects of shear stress on 3-D human mesenchymal stem cell construct development in a perfusion bioreactor system: Experiments and hydrodynamic modeling. Biotechnol Bioeng.

[23] Tjabringa GS, Vezeridis PS, Zandieh-Doulabi B (2006). Polyamines modulate nitric oxide production and cox-2 gene expression in response to mechanical loading in human adipose tissue-derived mesenchymal stem cells. Stem Cells.

[24] Li YJ, Batra NN, You L (2004). Oscillatory fluid flow affects human marrow stromal cell proliferation and differentiation. J Orthop Res.

[25] Riddle RC, Taylor AF, Rogers JR (2007). Atp release mediates fluid flow-induced proliferation of human bone marrow stromal cells. J Bone Miner Res.

[26] Riddle RC, Taylor AF, Genetos DC (2006). Map kinase and calcium signaling mediate fluid flow-induced human mesenchymal stem cell proliferation. Am J Physiol Cell Physiol.

[27] Singla V, Reiter JF (2006). The primary cilium as the cell's antenna: Signaling at a sensory organelle. Science.

[28] Praetorius HA, Spring KR (2005). A physiological view of the primary cilium. Annu Rev Physiol.

[29] Lee KL, Hoey DA, Jacobs CR (2012). Primary cilia-mediated mechanotransduction in bone. Clin Rev Bone Miner Metab.

[30] Hoey DA, Downs ME, Jacobs CR (2012). The mechanics of the primary cilium: An intricate structure with complex function. J Biomech.

[31] Ecker A Flimmerbewegung im Gehörorgan von Petromyzon marinus. Arch Anat Physiol Wiss Med.

[32] Zimmerman KW (1898). Beitrage zur Kenntniss einiger Drusen und Epithelien. Arch Mikrosk Anat.

[33] Hildebrandt F, Benzing T, Katsanis N (2011). Ciliopathies. N Engl J Med.

[34] Berbari NF, O'Connor AK, Haycraft CJ (2009). The primary cilium as a complex signaling center. Curr Biol.

[35] Praetorius HA, Spring KR (2001). Bending the MDCK cell primary cilium increases intracellular calcium. J Membr Biol.

[36] Nauli SM, Alenghat FJ, Luo Y (2003). Polycystins 1 and 2 mediate mechanosensation in the primary cilium of kidney cells. Nat Genet.

[37] Abdul-Majeed S, Moloney BC, Nauli SM (2012). Mechanisms regulating cilia growth and cilia function in endothelial cells. Cell Mol Life Sci.

[38] Masyuk AI, Masyuk TV, Splinter PL (2006). Cholangiocyte cilia detect changes in luminal fluid flow and transmit them into intracellular Ca^2+^ and CAMP signaling. Gastroenterology.

[39] Masyuk AI, Gradilone SA, Banales JM (2008). Cholangiocyte Primary cilia are chemosensory organelles that detect biliary nucleotides via P2Y12 purinergic receptors. Am J Physiol Gastrointest Liver Physiol.

[40] Shao YY, Wang L, Welter JF (2012). Primary cilia modulate Ihh signal transduction in response to hydrostatic loading of growth plate chondrocytes. Bone.

[41] Malone AM, Anderson CT, Tummala P (2007). Primary cilia mediate mechanosensing in bone cells by a calcium-independent mechanism. Proc Natl Acad Sci USA.

[42] Hoey DA, Kelly DJ, Jacobs CR (2011). A role for the primary cilium in paracrine signaling between mechanically stimulated osteocytes and mesenchymal stem cells. Biochem Biophys Res Commun.

[43] Kwon RY, Temiyasathit S, Tummala P (2010). Primary cilium-dependent mechanosensing is mediated by adenylyl cyclase 6 and cyclic AMP in bone cells. FASEB J.

[44] Tummala P, Arnsdorf EJ, Jacobs CR (2010). The role of primary cilia in mesenchymal stem cell differentiation: A pivotal switch in guiding lineage commitment. Cell Mol Bioeng.

[45] Xiao ZS, Quarles LD (2010). Role of the polycytin-primary cilia complex in bone development and mechanosensing. Ann N Y Acad Sci.

[46] Xiao Z, Zhang S, Cao L (2010). Conditional disruption of Pkd1 in osteoblasts results in osteopenia due to direct impairment of bone formation. J Biol Chem.

[47] Temiyasathit S, Tang WJ, Leucht P (2012). Mechanosensing by the primary cilium: Deletion of Kif3A reduces bone formation due to loading. PLoS One.

[48] Bianco P, Riminucci M, Gronthos S (2001). Bone marrow stromal stem cells: Nature, biology, and potential applications. Stem Cells.

[49] Jaiswal N, Haynesworth SE, Caplan AI (1997). Osteogenic differentiation of purified, culture-expanded human mesenchymal stem cells in vitro. J Cell Biochem.

[50] Jacobs CR, Yellowley CE, Davis BR (1998). Differential effect of steady versus oscillating flow on bone cells. J Biomech.

[51] Taulman PD, Haycraft CJ, Balkovetz DF (2001). Polaris, a protein involved in left-right axis patterning, localizes to basal bodies and cilia. Mol Biol Cell.

[52] Nowlan NC, Sharpe J, Roddy KA (2010). Mechanobiology of embryonic skeletal development: Insights from animal models. Birth Defects Res C Embryo Today.

[53] Lee HW, Kim SY, Kim AY (2009). Adiponectin stimulates osteoblast differentiation through induction of COX2 in mesenchymal progenitor cells. Stem Cells.

[54] Zhang X, Schwarz EM, Young DA (2002). Cyclooxygenase-2 regulates mesenchymal cell differentiation into the osteoblast lineage and is critically involved in bone repair. J Clin Invest.

[55] Forwood MR (1996). Inducible cyclo-oxygenase (COX-2) mediates the induction of bone formation by mechanical loading in vivo. J Bone Miner Res.

[56] Gerasimovskaya EV, Tucker DA, Weiser-Evans M (2005). Extracellular ATP-induced proliferation of adventitial fibroblasts requires phosphoinositide 3-kinase, Akt, mammalian target of rapamycin, and p70 S6 kinase signaling pathways. J Biol Chem.

[57] Lee DY, Li YS, Chang SF (2010). Oscillatory flow-induced proliferation of osteoblast-like cells is mediated by alphavbeta3 and beta1 integrins through synergistic interactions of focal adhesion kinase and Shc with phosphatidylinositol 3-kinase and the Akt/mTOR/p70S6K pathway. J Biol Chem.

[58] Cullen DM, Smith RT, Akhter MP (2001). Bone-loading response varies with strain magnitude and cycle number. J Appl Physiol.

[59] Plaisant M, Fontaine C, Cousin W (2009). Activation of Hedgehog signaling inhibits osteoblast differentiation of human mesenchymal stem cells. Stem Cells.

[60] Kiprilov EN, Awan A, Desprat R (2008). Human embryonic stem cells in culture possess primary cilia with Hedgehog signaling machinery. J Cell Biol.

[61] Whitfield JF, Chakravarthy BR (2009). The neuronal primary cilium: Driver of neurogenesis and memory formation in the hippocampal dentate gyrus?. Cell Signal.

[62] Clement CA, Kristensen SG, Mollgard K (2009). The primary cilium coordinates early cardiogenesis and hedgehog signaling in cardiomyocyte differentiation. J Cell Sci.

[63] Nakamura T, Aikawa T, Iwamoto-Enomoto M (1997). Induction of osteogenic differentiation by Hedgehog proteins. Biochem Biophys Res Commun.

[64] James AW, Leucht P, Levi B (2010). Sonic Hedgehog influences the balance of osteogenesis and adipogenesis in mouse adipose-derived stromal cells. Tissue Eng Part A.

[65] Murakami M, Ichisaka T, Maeda M (2004). Mtor is essential for growth and proliferation in early mouse embryos and embryonic stem cells. Mol Cell Biol.

[66] Shaw RJ, Cantley LC, Ras (2006). PI(3)K and mTOR signalling controls tumour cell growth. Nature.

[67] Boehlke C, Kotsis F, Patel V (2010). Primary cilia regulate mTORC1 activity and cell size through Lkb1. Nat Cell Biol.

[68] Shillingford JM, Murcia NS, Larson (2006). CH, The Mtor pathway is regulated by polycystin-1, and its inhibition reverses renal cystogenesis in polycystic kidney disease. Proc Natl Acad Sci USA.

[69] Weimbs T (2007). Polycystic kidney disease and renal injury repair: Common pathways, fluid flow, and the function of polycystin-1. Am J Physiol Renal Physiol.

[70] Litzenberger JB, Kim JB, Tummala P (2010). Beta1 integrins mediate mechanosensitive signaling pathways in osteocytes. Calcif Tissue Int.

[71] Young SRL, Gerard-O'Riley R, Kim JB (2009). Focal adhesion kinase is important for fluid shear stress-induced mechanotransduction in osteoblasts. J Bone Miner Res.

[72] McGlashan SR, Jensen CG, Poole CA (2006). Localization of extracellular matrix receptors on the chondrocyte primary cilium. J Histochem Cytochem.

[73] Praetorius HA, Frokiaer J, Praetorius J (2002). beta 1-integrin is expressed on the primary cilia of MDCK cells. J Am Soc Nephrology.

