# Supplementary figures and images for: Primary Cilia-Mediated Mechanotransduction in Human Mesenchymal Stem Cells
Source: Stem Cells. 2012 Sep 11;30(11):2561–70. doi: 10.1002/stem.1235 (PMC3533782; doi:10.1002/stem.1235)

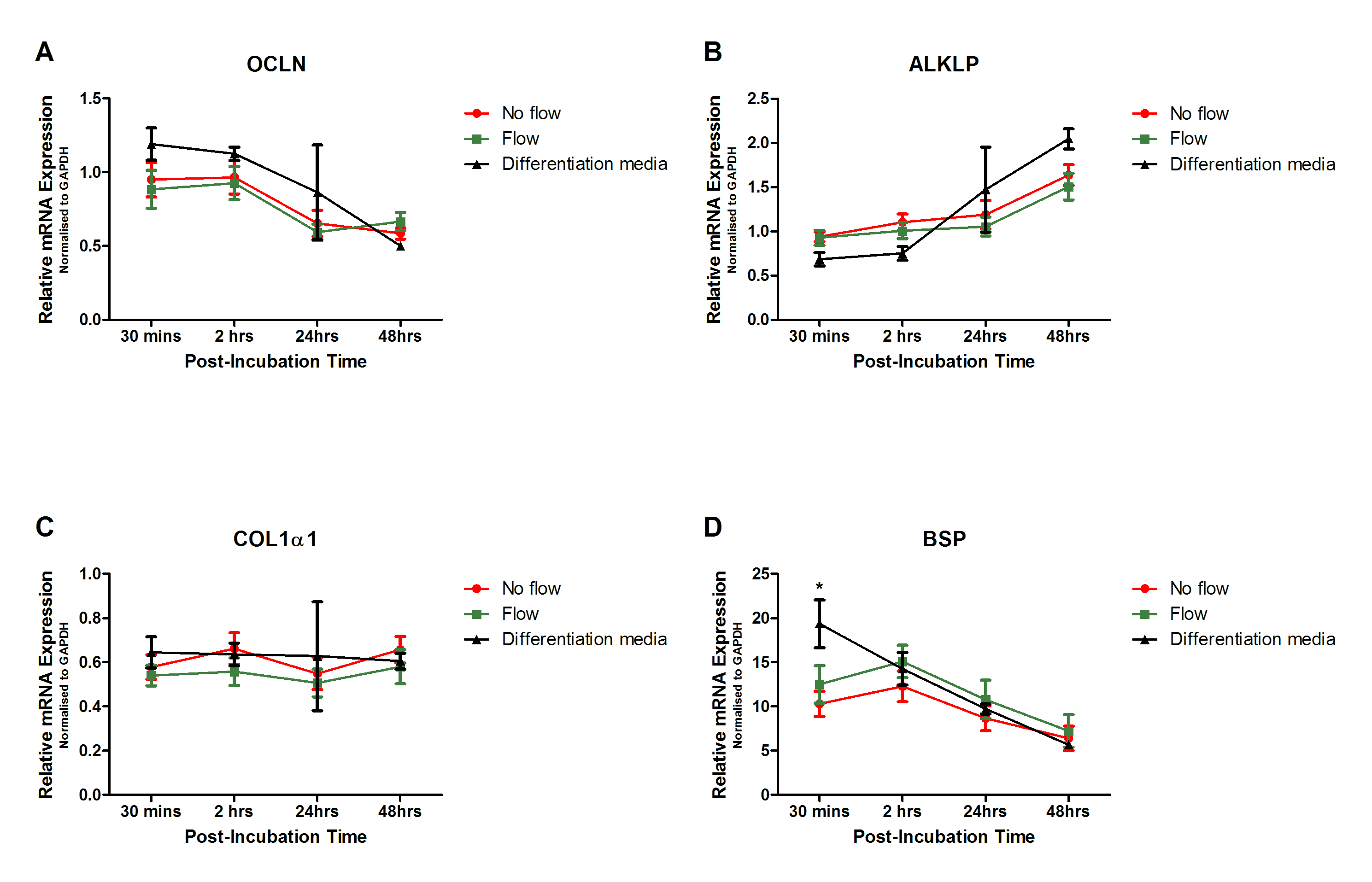

Supplement: Supplementary file 1 [file stem0030-2561-SD1.tif]
